# Bald eagle mortality and nest failure due to clade 2.3.4.4 highly pathogenic H5N1 influenza a virus

**DOI:** 10.1038/s41598-023-27446-1

**Published:** 2023-01-05

**Authors:** Nicole M. Nemeth, Mark G. Ruder, Rebecca L. Poulson, Robert Sargent, Shawnlei Breeding, Meaghan N. Evans, Jared Zimmerman, Rebecca Hardman, Mark Cunningham, Samantha Gibbs, David E. Stallknecht

**Affiliations:** 1 Southeastern Cooperative Wildlife Disease Study, College of Veterinary Medicine, University of Georgia, Athens, GA 30602 USA; 2 Department of Pathology, College of Veterinary Medicine, University of Georgia, Athens, GA 30602 USA; 3 Georgia Department of Natural Resources, Wildlife Resources Division, Georgia, Forsyth, GA 31029 USA; 4 Audubon Center for Birds of Prey, 1101 Audubon Way, Maitland, FL 32751 USA; 5 Fish and Wildlife Research Institute, Florida Fish and Wildlife Conservation Commission, Gainesville, FL 32601 USA; 6 Wildlife Health Office, National Wildlife Refuge System, United States Fish and Wildlife Service, Chiefland, FL 32626 USA

**Keywords:** Ecology, Microbiology, Diseases

## Abstract

The bald eagle (*Haliaeetus leucocephalus*) is a culturally and ecologically vital species in North America that embodies conservation success but continues to face threats that include emerging pathogens. The introduction of A/goose/Guangdong/1/1996 lineage highly pathogenic (HP) clade 2.3.4.4b H5N1 influenza A virus (IAV) in North America in late 2021 resulted in high rates of mortality among bald eagles. Here we show an alarming rate of bald eagle nest failure and mortality attributed to HP IAV. We documented fatal, systemic HP IAV infection in breeding adult and nestling bald eagles along the southeastern U.S. coast. Concurrently, annual bald eagle nest surveys in Georgia and Florida revealed a precipitous drop in success in coastal counties compared with previous years, portending negative impacts on population recruitment. As an apex predator and efficient scavenger, it is likely that bald eagles become infected through consumption of infected waterfowl. These results and similar reports of raptor mortality in Europe, Asia, and Africa, indicate a clear threat to raptor health. The possible long-term persistence of HP H5N1 IAV in North America poses an impending threat to bald eagle populations not only related to direct mortality but also decreased recruitment and warrants continued efforts to understand these potential impacts.

## Introduction

The bald eagle (*Haliaeetus leucocephalus*) is a culturally and ecologically vital species that embodies conservation success. The breeding range of the bald eagle encompasses aquatic habitats coast-to-coast across much of Canada and portions of the United States, especially along the Atlantic Coast from South Carolina to Florida, the Chesapeake Bay region and north to Maine, as well as the Great Lakes region, Pacific Northwest, and along portions of the Rocky Mountains and Gulf Coast. Species conservation concerns remain, however, despite a substantial increase in the numbers of productive bald eagle nesting pairs, numbers of individuals, and area of geographic distribution in the contiguous 48 U.S. states since the species was downlisted from Endangered to Threatened under the Endangered Species Act in 1995^1^.

Ongoing threats to the continued recovery of the bald eagle often are anthropogenic and include exposure to environmental contaminants (e.g., lead and anticoagulant rodenticides)^2,3^, trauma from vehicular, wind turbine, or powerline strikes, and direct persecution^1,4^. Historic threats include DDT (dichloro-diphenyl-trichloroethane) metabolites in pesticides that drastically reduced reproductive success beginning in the 1950s, leading to near extinction in the contiguous U.S.^1,5^. While most of the contemporary threats are widespread and commonly reported, many can be mitigated through enforcement of legislation, conservation management, and educational outreach initiatives. The effectiveness of such loss-reduction strategies may decrease in the face of stochastic epidemiological threats such as emerging unpredictable, unmanageable, or unavoidable infectious disease outbreaks.

During the past 20 years, highly pathogenic (HP) A/goose/Guangdong/1/1996 lineage (Gs/GD) H5 influenza A virus (IAV) outbreaks in Europe, Asia, and Africa have increased in frequency and severity in a variety of wild bird species^6,7^ In December 2021, Gs/GD H5N1 IAV (hereafter, HP IAV) was introduced to eastern North America from Europe^8^. In North America, the virus initially was detected in a variety of wild avian species in Newfoundland and Labrador, Canada, which was quickly followed by detections in migratory waterfowl in southern breeding grounds in the United States. To date, detections continue throughout much of North America^9–11^. Mortalities attributed to HP IAV have now been documented in a diversity of wild bird species. Taxa most commonly infected have included waterfowl, shorebirds, raptors and scavenging birds^10–14^.

**Figure 1:**
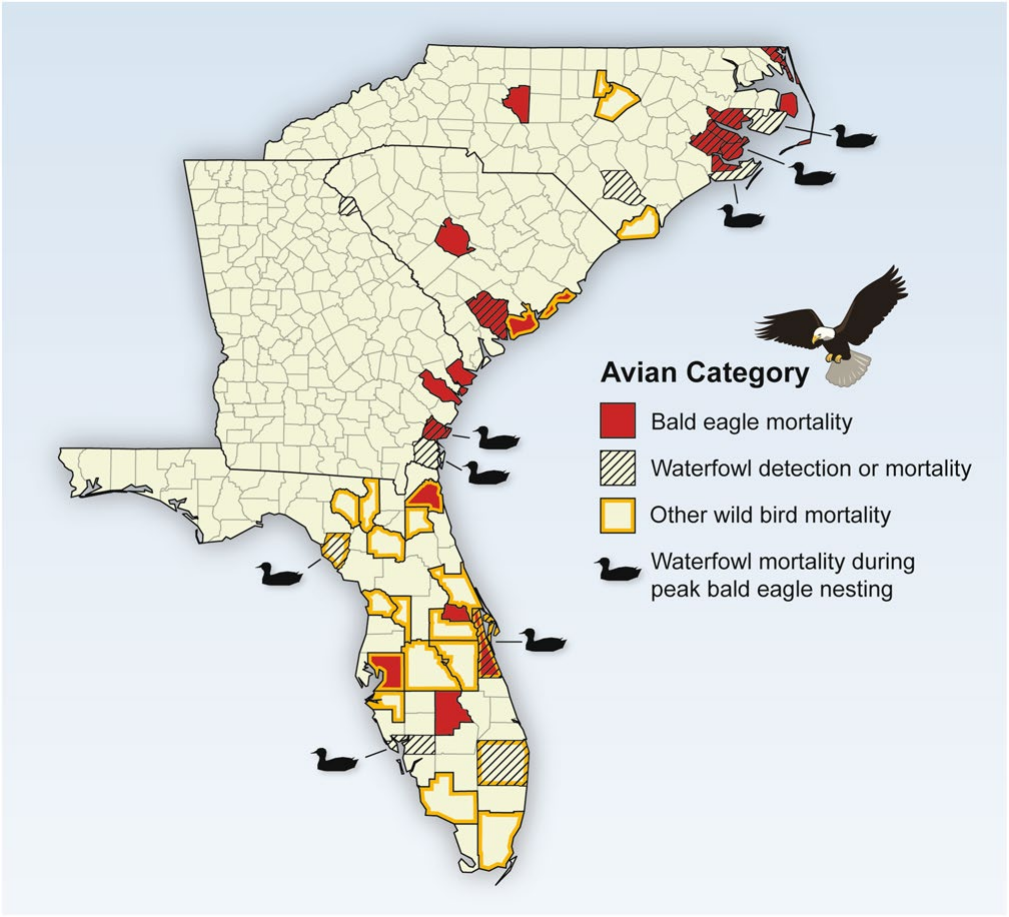
Regional highly pathogenic influenza. A virus in wild birds during Spring 2022 in the southeastern USA. Counties in North Carolina, South Carolina, Georgia, and Florida where H5 influenza A virus (IAV) (A/goose/Guangdong/1/1996 lineage HP clade 2.3.4.4b H5N1) was detected in wild birds during Spring 2022, as reported by the U.S. Department of Agriculture (USDA; https://www.aphis.usda.gov/aphis/ourfocus/animalhealth/animal-disease-information/avian/avian-influenza/hpai-2022/2022-hpai-wild-birds; website accessed July 22, 2022), with additional detections by the Southeastern Cooperative Wildlife Disease Study, University of Georgia. Detections were made through wild bird mortality investigations, or USDA surveillance of hunter-harvested waterfowl. The map shows counties with bald eagle H5 IAV mortality (red), as detected by SCWDS and reported on the USDA website from January 1 through May 15, 2022; detections of H5 IAV in dead or hunter-harvested waterfowl (hash lines) as reported on USDA website from January 1 through April 1, 2022; H5 IAV detection or mortality in wild bird species other than bald eagle or waterfowl (yellow border) as detected by SCWDS and reported on USDA website from January 1 through May 15, 2022. Duck silhouettes identify locations with confirmed H5 IAV waterfowl mortality coincident with peak bald eagle nesting activity from January 1 through April 1, 2022, as detected by SCWDS and reported on USDA website^13^.

Since January 2022, HP IAV-related mortality in bald eagles has been confirmed in 136 individuals collected from 24 U.S. states (as of June 10, 2022), including many along the southern Atlantic coast (Fig. 1)^10^. Some of these eagles that were observed while still alive exhibited distress, head shaking, ataxia, inability to fly, paralysis, and/or lethargy (Supplementary videos 1 and 2). Pathology often was severe and included multi-organ necrosis and brain inflammation similar to recent outbreaks of HP IAV H5N8 and H5N1 in Europe^13–15^.

**Figure 2:**
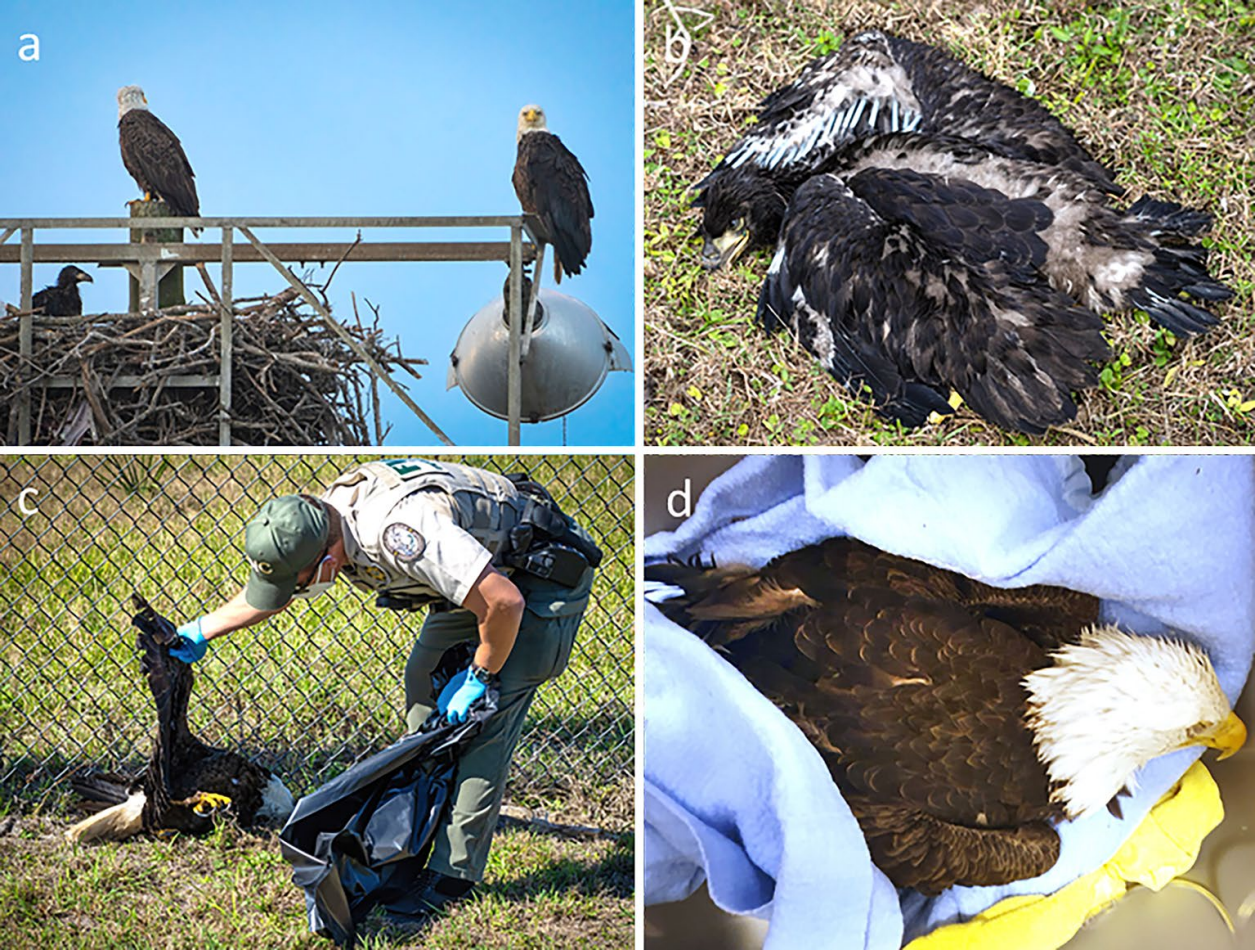
Images illustrating severe disease and mortality from H5 influenza A virus infection in bald eagles (*Haliaeetus leucocephalus*). (**a**) An attentive nesting pair of bald eagles (nest BE106) with an apparently healthy nestling on February 17, 2022, in Brevard County, Florida (photo by Bob Glover). (**b**) Nestling bald eagle from the same nest (BE106) taken February 18, 2022, after being found dead under the nest and later confirmed to have died from H5 IAV infection (photo by Bob Glover). (**c**) A Florida Fish and Wildlife Conservation Commission law enforcement officer collecting the carcass of the adult female from the same nest (BE106) after being found dead under the nest on February 24, 2022, after succumbing to H5 IAV infection (Photo by Bob Glover). (**d**) Adult bald eagle from Davidson County, North Carolina infected with H5 IAV and presented to a wildlife rehabilitation facility with severe neurologic signs (e.g., severe lethargy, seizures, partial paralysis); the eagle was administered oxygen, subcutaneous fluids, antibiotics, diazepam (valium®) but died the following morning (photograph by Jackie Schaible).

In Florida, Georgia and South Carolina, early stages of the outbreak temporally coincided with the bald eagle nesting season. This region contains large and productive populations of breeding eagles that directly contribute to the long-term viability of the species within the Atlantic Flyway^16–18^. Breeding pairs establish nesting territories in close proximity to bodies of water containing high-quality prey such as fish or waterfowl^1,19,20^. Waterfowl commonly occur in these habitats and represent the primary reservoir for IAV^6^. In at least four nesting territories, adult and young eagles displayed abnormal behaviors, often interpreted as neurologic signs, prior to being found dead near active nests, while others conspicuously fell from nests. These mortalities were confirmed as HP IAV infections. At two of these nest sites, one or both parents were unaccounted for and later determined to have died from HP IAV (Fig. 2a,b,c,d). These findings have prompted concerns for potentially longer-term and more pervasive effects of this outbreak in terms of population health.

## Results

### Eagle morbidity and mortality

Overlapping with the time period of nest observations (from January 25 to March 17, 2022) bald eagles received for postmortem evaluation from four southeastern states (North Carolina, South Carolina, Georgia and Florida) were diagnosed with HP avian influenza through gross and histopathology when possible, and real-time reverse transcription polymerase chain reaction (rRT-PCR). During this time, 22 bald eagles (14 adult eagles and 8 nestlings) were diagnosed with fatal highly pathogenic influenza based on pathology, including some combination of splenic, hepatic, pancreatic, adrenal, and myocardial degeneration and/or necrosis, encephalitis + / − neuronal necrosis, and nephritis, and laboratory confirmation of HP IAV in oropharyngeal and cloacal swabs tested at NVSL. Among these, five actively breeding adult eagles either were observed to fall from the nest or were found beneath a nest; two of the adults were observed actively nesting 1–2 days before death. Suspected to be a sequela to HP IAV infection, concurrent trauma, evidenced by intracoelomic hemorrhage, pulmonary, hepatic, renal, intramuscular, and/or intracranial hemorrhage, ruptured or lacerated internal organs (e.g., liver, heart, kidney), commonly was observed (12/22; 54.5%) (Supplemental Table 1 and supplemental Figure 1).

### Georgia nest observations

**Figure 3:**
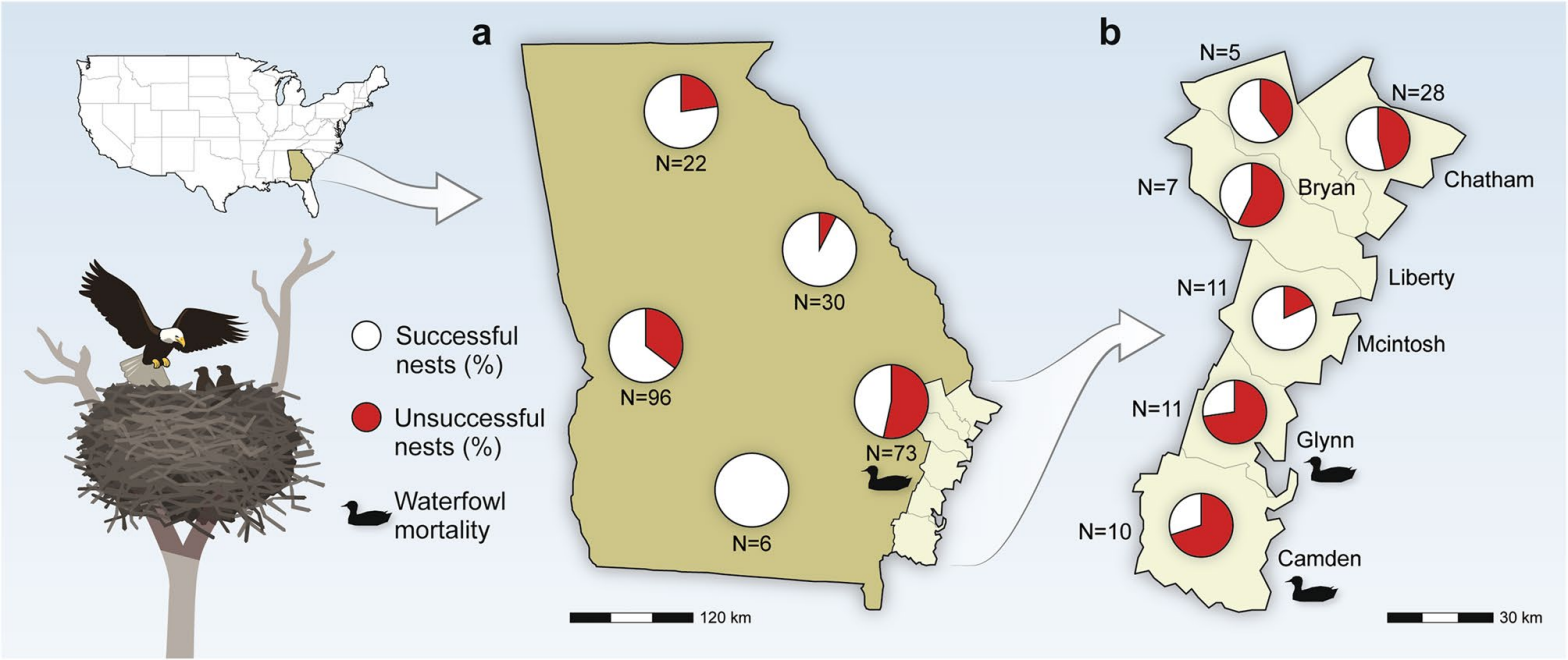
Reproductive indices for nesting bald eagles in Georgia, USA in 2022. (**a**) Regional bald eagle percent nest success in Georgia during 2022. (**b**) Bald eagle nest success in Atlantic coastal counties of Georgia during 2022. Aerial nest surveys are performed annually during March by the Georgia Department of Natural Resources. *N* = number of occupied nest territories surveyed.

**Figure 4:**
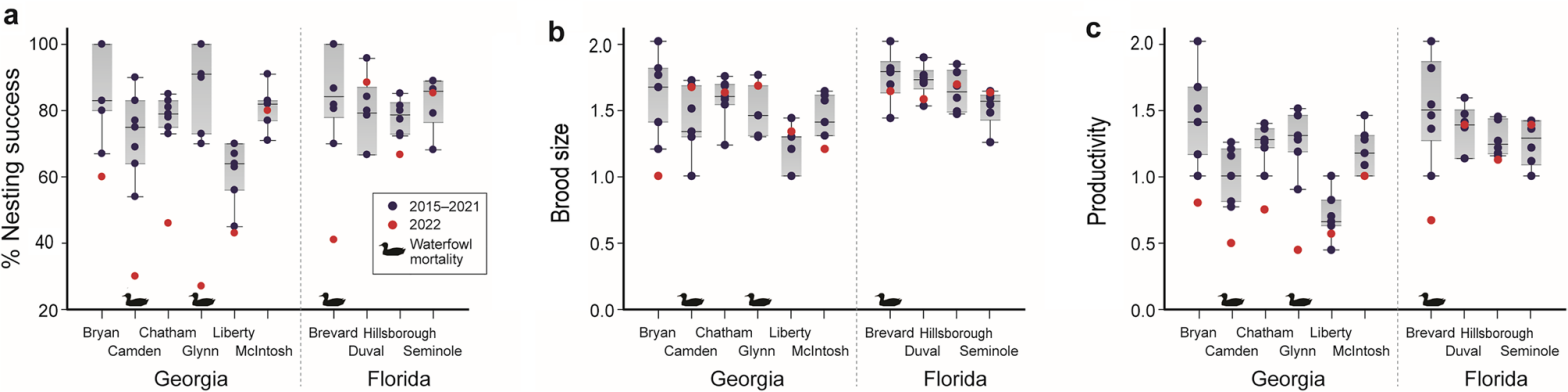
Bald eagle nest metrics (2015–2022) in selected counties of Georgia and Florida, USA where H5 highly pathogenic influenza A virus mortality in bald eagles was confirmed January—April 2022. (**a**) Bald eagle nest success. (**b**) Brood size (mean fledged bald eagles/successful nest). (**c**) Productivity (mean fledged bald eagles/occupied territory (GA) or /occupied nest (FL)). Red dots represent values for 2022. Duck symbols represent those counties with documented mortality among overwintering waterfowl (e.g., lesser scaup).

In Georgia, a 2022 aerial statewide survey revealed 47% nest success for bald eagles in the coastal region, which is about 30% below the average for the region. In contrast, nest success in the four inland regions ranged from approximately 65% to 100%, consistent with previous years (Supplemental Table 2). Within the coastal region in 2022, most (72 of 73) of the monitored eagle nests were located in six counties, and county-specific nest success metrics ranged from 27 to 81% (Fig. 3a,b and Supplemental Table 2). With the exception of McIntosh County, nest success was lower than the mean county nest success rate for years 2015–2021 and below observed rates for any of the previous seven years (Fig. 4a). The greatest reductions in nest success metrics were observed in Camden and Glynn counties, where 2022 rates dropped 43% and 62%, respectively, from historical (2015–2021) mean levels. There was much less variation within metrics of individual nesting outcomes from these counties, and in four counties that included Camden and Glynn; brood size estimates from 2022 were within the 2015–2021 range (sampled in January–February and March–April) and comparable to the mean for those years (Fig. 4b and Supplemental Table 2). Nest productivity followed the patterns observed for nest success, with reductions observed in the same four counties in 2022 (Fig. 4c). In Camden and Glynn counties, comparisons between 2015–2021 and 2022 revealed decreases in annual productivity from 1 to 0.5 and 1.3 to 0.45, respectively. The 2022 estimates of these metrics may in fact represent an overestimate of nesting outcomes, as Georgia nests were deemed successful on the second aerial survey (i.e., conducted in March) if they had eaglets in advanced development or, in some instances, in younger stages. Additionally, HP IAV-associated avian mortalities in many wild birds, including bald eagles, continued past nest monitoring and fledging of the latter; thus, some of these nests may have failed post-survey data collection.

### Florida nest observations

In Florida, ground-based bald eagle nest surveys conducted in four selected coastal counties revealed similar (to Georgia) county-level variations in reproductive metrics from the 2022 breeding season. These counties were selected based on locations of early detections of HP IAV-associated bald eagle mortalities within the outbreak trajectory, resulting in intensified surveillance efforts. A decrease in nest success was observed in Brevard and Hillsborough counties in 2022 compared to those observed in 2015–2021 (Supplementary Table 3). When contrasted with the mean metrics reported from the preceding 6-year observational period, 2022 nest success in these two counties, dropped from 86.5 to 41.0% and 78.3 to 66.7%, respectively (Fig. 4a). Consistent with the Georgia data, there were no apparent reductions in the 2022 brood size observed in select Florida counties (Fig. 4b), but a reduction was observed in annual productivity (Fig. 4c). This reduction was most apparent in Brevard County, where 0.67 eaglets fledged per occupied nest in 2022 compared to a historical average of 1.52 (Supplemental Table 3).

## Discussion

Bald eagle reproduction was most impacted in counties where HP IAV-associated mortality in lesser scaup (*Aythya affinis*) also was detected (Figs. 1, 3 and 4)^10^. This waterfowl species is one of the most abundant and widespread of North American ducks, and has a wintering range that includes southeastern Atlantic coastal states, including the Carolinas south to Florida^21^. Although no reliable county-level counts of the number of lesser scaup affected by HP IAV are available, observational estimates indicate mortalities likely were in the thousands. These high numbers of mortalities coincided with the eagle breeding season, a time of increased food demand alongside the tendency of bald eagles to scavenge debilitated prey and readily accessible carcasses^1^. These combined environmental and biological features likely facilitated a perfect epizootic storm elevating HP IAV transmission to bald eagles. Pathology in infected eagles is consistent with acute death; in addition, lesions consistent with severe, blunt-force trauma were evident in many eagles diagnosed with HP avian influenza, and were suspected (and sometimes observed) to be due to ground impact after falling from branches or nests.

The impacts of infection manifested beyond the scale of individual eagles, and directly affected population recruitment dynamics through elevated rates of reproductive failures. Although these patterns thus far have only been observed locally, it is important to note the broad distribution of detected eagle mortalities from HP IAV throughout North America, including as far north as Alaska^10^. These results represent early warning of an emerging threat to avian populations across North America and beyond^11^, especially to waterfowl and those ecologically tied to them^15^, and further research is urgently needed to optimize adaptive management strategies to offset these multi-scale losses.

The long-term effects, including possible population-level impacts, of this HP IAV outbreak on bald eagles and other wild bird species remain to be seen^6^. If HP IAV persists in North America, the proclivity of bald eagles for nesting near and along bodies of water cohabited by waterfowl may be to their detriment. These observed impacts of HP IAV on bald eagles represent an early horizon scan of an emerging threat to populations of predatory and scavenging birds across North America. In Florida, bald eagles were one of the earliest species in which HP IAV infection was documented, and are an over-represented species among those confirmed with HP IAV infections in the current outbreak. Further, bald eagles were detected with HP IAV in areas that did not correspond to waterfowl congregations^10^. Thus, as has been suggested for common buzzards (*Buteo buteo*) and white-tailed sea eagles (*Haliaeetus albicilla*) infected with HP H5N8 IAV in Europe^13,14^, bald eagles may serve as a sensitive ecological indicator species for this virus in North America, just as they have for environmental contaminants such as lead, mercury, and the more recently characterized aetokthonotoxin, an alkaloid toxin produced by the cyanobacterium *Aetokthonos hydrillicola*^22^. However, it is important to recognize that eagle nest success is not routinely monitored in many areas and without more comprehensive monitoring, future impacts will not be detected, quantified or (potentially) mitigated.

## Methods

### Sample collection and postmortem evaluation

Bald eagle carcasses, and/or oropharyngeal and cloacal swabs were collected in the field and submitted to the Southeastern Cooperative Wildlife Disease Study Research and Diagnostic Service. In some cases, live bald eagles were found moribund and transported to wildlife rehabilitation clinics and either died in transit or soon after arrival. Carcasses underwent postmortem evaluation, including gross and histopathology. Tissue samples [heart, brain, kidney, spleen, lung, adrenal gland, pancreas, liver, small and large intestine, and cloacal bursa (if present)] were fixed in 10% neutral buffered formalin and routinely processed for histopathology^23^ at the Athens Veterinary Diagnostic Laboratory. Histopathology was assessed by a board-certified veterinary pathologist.

### Additional bald eagle and waterfowl species mortality data

Data on wild bird deaths attributed to highly pathogenic influenza A viruses were retrieved from the U.S. Department of Agriculture, Animal and Plant Health Inspection Service website, at: https://www.aphis.usda.gov/aphis/ourfocus/animalhealth/animal-disease-information/avian/avian-influenza/hpai-2022/2022-hpai-wild-birds. These data are publicly available and include state, county, date detected, and species of individual birds that tested positive for HP IAV.

### Immunohistochemistry

Immunohistochemistry (IHC) for avian influenza virus was performed in select cases on brain, pancreas, spleen, liver, and/or adrenal gland at the Athens Veterinary Diagnostic Laboratory. IHC was performed on an automated stainer (Nemesis 3600, Biocare Medical). Polyclonal antiserum against influenza A virus was used as the primary antibody (ab155877, Abcam), diluted 1:3000, and incubated for 60 min at 37 °C with agent-positive control. Antigen retrieval was with Target Retrieval Solution (S2367, Dako) pH (10x) at 110 °C for 15 min. Enzyme blockage was via 3% H_2_O_2_ for 20 min (H324-500, Fisher Scientific); protein blockage was with Universal Blocking Reagent (10x) Power Block diluted at 1:10 for 5 min (HK085-5 K, BioGenex); link was by biotinylated rabbit anti-goat (BA-5000, Vector) at a 1:100 dilution for 10 min with 4 + streptavidin alkaline phosphatase label for 10 min (AP605H, BioCare Medical). Staining was with warp red chromogen kit for 5 min (WR8065, BioCare Medical). Known influenza A-virus positive control tissues were tested alongside each case.

### Polymerase chain reaction

Oropharyngeal and cloacal swabs from bald eagle carcasses were pooled for each individual eagle and tested by real-time reverse transcription polymerase chain reaction (rRT-PCR). Briefly, swabs samples were extracted with the KingFisher magnetic particle processer using the MagMAX-96 AI/ND Viral RNA isolation Kit (Ambion/Applied Biosystems, Foster City, CA) following a modified MagMAX-S protocol^24^. Resultant nucleic acids were screened against primers specific for H5 IAV in rRT-PCR; samples that yielded a cycle threshold value < 40 were submitted to the National Veterinary Services Laboratory, Ames, Iowa for confirmation of H5 highly pathogenic influenza A virus.

### Bald eagle nest monitoring in Georgia, USA

From 2015 to 2017 and in 2022, the Georgia Department of Natural Resources conducted annual statewide surveys, primarily by helicopter, to assess nest success rates of bald eagles in 100% of known nest territories. From 2018–2020, 60–70% of known nest territories from the previous year’s surveys were monitored annually, including complete annual monitoring of the six coastal counties. In 2021, due to COVID-19 restrictions, only the six coastal counties were surveyed. Initial aerial surveys each nesting season were conducted between the first week of January and the first week of February, and sought to locate all active nests. Monitoring was performed according to the latitudinal gradient with regards to the timing of nesting cycles (i.e., eagles on the coast nest earlier and fledge young earlier than those in the mid-state, and much earlier than those nesting on reservoirs in the mountains). A nest was deemed occupied if it contained an adult eagle in an incubating posture, egg(s), eaglets, adult eagles engaged in nest affinity, or evidence of nest building and preparation (e.g., presence of fresh greenery, sticks with fresh breaks, or a new layer of nesting material^25^. These initial aerial surveys focused on nests that were active in the most previous survey, reports of potential new nest sites, and examination of sites having a high probability of supporting new nest territories.

The second round of aerial nest surveys took place from mid-March to early April, and were conducted to determine the reproductive outcome of the nests visited in January and February, as well as to visit newly reported nests. These follow-up flights aimed to maximize the probability that, if the nests in a survey route have not failed, they should contain eaglets that are approximately 80% of the age at first flight. Nests with eaglets of this age or older were considered successful as nestling mortality beyond this stage generally is low^26,27^. However, the timing of the commencement of nesting activities by eagle pairs within an ecoregion can vary by a few weeks. The second round of surveys confirmed that most of the nests previously determined as active had eaglets in advanced stages of development (i.e., 9–12 weeks of age), but occasionally included eaglets as young as 2–3 weeks. For these surveys, nests were recorded as successful if they contained eaglets during the follow-up flights or if eaglets were observed branching near the nests (i.e., successfully fledged), with percent nest success defined as the percentage of total occupied nests with successfully fledged eaglets. Nests were recorded as unsuccessful if eggs were still present or an adult exhibiting incubating posture was in the nest, if dead eaglets were observed, if they were empty and eaglets should have been present, or if either the nest or supporting tree had fallen. Mean annual productivity was calculated as the sum of fledglings produced divided by the sum of occupied nesting territories. Annual brood size was defined as the total count of fledglings per successful nests.

### Bald eagle nest monitoring in Florida, USA

From 2016–2022, Audubon EagleWatch, a community science program, monitored bald eagle nests across the state of Florida (https://cbop.audubon.org/conservation/about-eaglewatch-program); data from four coastal counties with known incidents of early outbreak stage bald eagle HPAI mortality were used in this study. Trained volunteers visited nests a minimum of every two weeks during the peak bald eagle nesting period (October 1-May 15) with a minimum monitoring period of 20 min at each nest per site visit. One to four volunteers monitor each nest to ensure a robust dataset ideally spanning a range of temporal observation periods.

Nests that remained inactive or failed by May 15 of each year were no longer monitored, while occupied nests were monitored until young were fully fledged. Observed nests were either viewable from publicly accessible sites or via private property with appropriate permission. Thus, efforts were most concentrated within urban and suburban areas, and monitored nests can vary each survey year. Nest observers recorded data from the ground beneath the nest and from a distance that does not disturb the eagles (approximately 100 m, with some variation based on the nesting eagle’s tolerance of human activity). Nesting data were submitted via a custom Survey123 form and includes nest identifier and location, nest status, date and time, count of observed adults and young, and number of young fledged and/or perished noted during each site visit. If available, additional information on nest relocation, substrate changes, disturbance issues, and any observed leg bands is included. Nest status was inactive if no eagles were observed, only one adult was consistently observed over repeated visits, or two non-breeding adults were observed but displayed no interest in utilizing the nest. Nests were deemed occupied if a pair of eagles was consistently seen at the nest and showed interest in using the nest.

At the end of each nesting season, the EagleWatch Program Manager reviewed, validated, and summarized all submitted observations. Metrics reported for the 2022 season were extracted from institutional database version 2022–05-19. Data quality control includes case-by-case exclusion of nests with insufficient monitoring efforts. Eaglets between 8–10 weeks of age were assumed to have successfully fledged if the nest was empty after this period^28^. If no young were successfully produced, occupied nests were given a final status of failed, while nests that fledged at least one chick were categorized as successful. As with Georgia, post-fledging mortality is not accounted for in these analyses. However, unlike for Georgia eagle surveys, any sick or injured eaglets that were collected from monitored sites and successfully rehabilitated (i.e., released back into the wild) were considered successfully fledged. If a rescued eaglet was unable to be released, the young was categorized as perished (i.e., removed from the wild population). As with Georgia, percent nest success in Florida was defined as the percentage of total occupied nests that successfully fledged an eaglet. Summary nesting metrics of annual brood size (mean fledglings per successful nests) and productivity (fledglings per occupied nests) similarly aligned with definitions used in the Georgia methods.

All methods were carried out in accordance with relevant guidelines and regulations, and were done so with approval of the University of Georgia Institutional Animal Care and Use Committee (AUP# A2020 11–010-A6) and USFWS Eagle Scientific Collecting Permit number MB49069B-0.

## Supplementary Information

Supplementary Information 1.

Supplementary Video 1.

Supplementary Video 2.

## Data Availability

The data that support the findings of this study are available from the corresponding authors on reasonable request.
