# Supplementary material for: Bald eagle mortality and nest failure due to clade 2.3.4.4 highly pathogenic H5N1 influenza a virus
Source: Sci Rep. 2023 Jan 5;13:191. doi: 10.1038/s41598-023-27446-1 (PMC9813463; doi:10.1038/s41598-023-27446-1)
Supplement: Supplementary file 1 — Supplementary Information 1. [file 41598_2023_27446_MOESM1_ESM.pdf]

SUPPLEMENTAL MATERIAL

Bald eagle mortality and nest failure due to clade 2.3.4.4 highly pathogenic H5N1 influenza A virus

Nicole M. Nemeth[1,2,*], Mark G. Ruder[1], Rebecca L. Poulson[1], Robert Sargent[3], Shawnlei Breeding[4], Meaghan N. Evans[5], Jared Zimmerman[5], Rebecca Hardman[5], Mark Cunningham[5], Samantha Gibbs[6] & David E. Stallknecht[1]

[1]Southeastern Cooperative Wildlife Disease Study, College of Veterinary Medicine, University of Georgia, Athens, GA 30602, USA
[2]Department of Pathology, College of Veterinary Medicine, University of Georgia, Athens, GA 30602, USA
[3]Wildlife Resources Division, Georgia Department of Natural Resources, Forsyth, GA 31029, USA
[4]Audubon Center for Birds of Prey; 1101 Audubon Way, Maitland, FL 32751, USA
[5]Florida Fish and Wildlife Conservation Commission, Fish and Wildlife Research Institute, Gainesville, FL 32601, USA
[6]Wildlife Health Office, National Wildlife Refuge System, United States Fish and Wildlife Service, Chiefland, FL 32626, USA
[*]Corresponding author. Email: nmnemeth@uga.edu

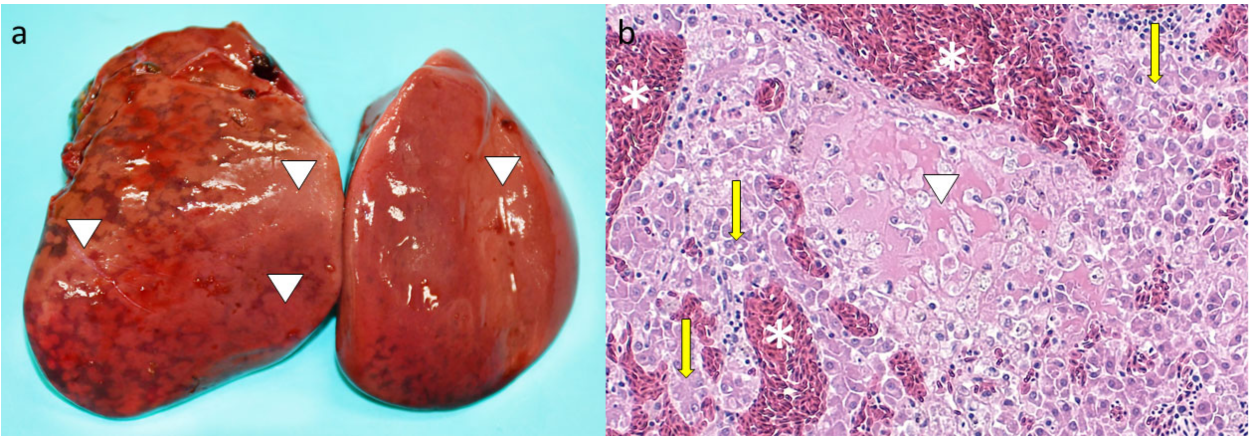

Supplemental Figure 1 Lesions characteristic of highly pathogenic influenza A virus in bald eagles. a, Gross liver lesions of necrosis (pallor; arrowheads) and congestion (dark red areas; arrows) associated with HP IAV infection in a bald eagle found at Savannah Golf Club in Chatham County, Savannah, GA. This liver corresponds to bald eagle W22-194B in Supplemental table 1. **b,** Microscopic liver lesions that consist of foci of liquefactive necrosis (white arrowhead), hepatocellular necrosis and degeneration (yellow arrows), and marked congestion (asterisks) associated with HP IAV in a bald eagle from High Rock Lake, Davidson County, NC. This liver corresponds to bald eagle W22-186 in Supplemental table 1.

ID	State	County	Date died	Age	Sex	Clinical signs	Lesions	Trauma
180-22	NC	Beaufort	March 8	M	F-U	Found dead	NHP	No
229-22	NC	Craven	March 12	M	M-U	Neurologic	Yes	No
227-22	NC	Currituck	March 8	M	F-U	Neurologic	Yes	Yes
140-22	NC	Dare	Feb. 22	M	M-U	Found dead	Yes	No
186-22	NC	Davidson	March 8	M	M-U	Neurologic	Yes	No
228-22	NC	Pamlico	March 17	M	F-U	Neurologic	Yes	No
170-22A	SC	Charleston	March 4	N	F-AN	None reported	Yes	Yes
170-22B	SC	Charleston	March 4	N	F-AN	None reported	Yes	Yes
218-22	SC	Colleton	March 17	N	U-AN	None reported	NAP	Unknown
205-22	SC	Lexington	March 1	N	F-AN	None reported	Yes	Yes
194-22A	GA	Glynn	March 8	M	M-U	Found dead	Yes	Yes
194-22B	GA	Chatham	March 14	M	F-U	Found dead	Yes	Yes
202-22	GA	Liberty	March 1	N	M-AN	Found dead	Yes	Yes
114-22	FL	Brevard	Feb. 16	M	F-AN	Neurologic	Yes	Yes
134-22	FL	Brevard	Feb. 12	M	M-U	None reported	Yes	Yes
142-22	FL	Brevard	Feb. 18	N	M-AN	Found dead	Yes	Yes
153-22A	FL	Brevard	Feb. 24	M	F-AN	Found dead	NHP	Yes
153-22B	FL	Brevard	Feb. 18	N	F-AN	Found dead	Yes	Yes
195-22	FL	Duval	March 13	N	M-AN	Neurologic	Yes	No
189-22	FL	Seminole	March 3	M	F-U	Found dead	NHP	No
191-22	FL	Seminole	March 8	M	M-U	Neurologic	Yes	No
269-22	FL	Hillsborough	Jan. 25	M	M-U	Neurologic	Yes	No

Supplemental Table 1 Case summary of bald eagles diagnosed with highly pathogenic avian influenza during January-March, 2022 at the Southeastern Cooperative Wildlife Disease Study. Age of eagles was either mature (M) or nestling (N); sex was male (M-U) or female (F-U) of unknown breeding status, male (M-AN) or female (F-AN) actively nesting (i.e., observed in nest or nestling age); or unknown sex actively nesting (U-AN). Five actively breeding mature eagles either were observed to fall from or were found beneath their nest. Clinical signs attributed to neurologic disease included inability to fly, distress, head shaking, ataxia, seizures, partial paralysis, lethargy, lack of awareness of surroundings. Lesions attributable to HPAIV included multi-organ necrosis (e.g., liver, spleen, pancreas, adrenal gland, heart), nephritis, and encephalitis (sometimes with neuronal necrosis). Gross and microscopic lesions attributable to trauma included intracoelomic hemorrhage, pulmonary, hepatic, renal, intramuscular, and/or intracranial hemorrhage, ruptured or lacerated internal organs (e.g., liver, heart, kidney). NAP = no autopsy performed; NHP = no histopathology performed (due to advanced decomposition).

County	Nest metrics	2015	2016	2017	2018	2019	2020	2021	7 yr avg	2022
Bryan	Number occupied territories	5	3	6	6	6	6	2	4.86	5
	% nest success	80.0	100.0	83.0	83.0	67.0	83.0	100.0	85.10	60.0
	Brood size	1.40	1.66	1.40	1.80	1.75	1.20	2.00	1.60	1.00
	Productivity	1.40	1.66	1.16	1.50	1.40	1.00	2.00	1.45	0.80
Camden	Number occupied territories	12	13	13	12	10	11	13	12.00	9
	% nest success	83.0	69.0	54.0	75.0	90.0	64.0	77.0	73.10	30.0
	Brood size	1.50	1.67	1.71	1.33	1.33	1.29	1.00	1.40	1.66
	Productivity	1.25	1.15	1.00	1.00	1.20	0.81	0.77	1.03	0.50
Chatham	Number occupied territories	24	24	23	26	26	23	26	24.57	28
	% nest success	75.0	79.0	78.0	81.0	85.0	83.0	73.0	79.10	46.0
	Brood size	1.67	1.53	1.23	1.57	1.59	1.68	1.74	1.57	1.62
	Productivity	1.25	1.21	1.00	1.27	1.35	1.39	1.27	1.25	0.75
Glynn	Number occupied territories	11	10	11	11	10	11	10	10.57	11
	% nest success	100.0	70.0	91.0	100.0	100.0	73.0	90.0	89.10	27.0
	Brood size	1.45	1.29	1.30	1.45	1.30	1.75	1.67	1.46	1.67
	Productivity	1.27	0.90	1.18	1.45	1.30	1.27	1.50	1.27	0.45
Liberty	Number occupied territories	10	9	11	11	10	9	8	9.71	7
	% nest success	70.0	67.0	45.0	64.0	70.0	56.0	63.0	62.10	43.0
	Brood size	1.43	1.00	1.00	1.29	1.00	1.20	1.00	1.13	1.33
	Productivity	1.00	0.66	0.45	0.82	0.70	0.66	0.63	0.70	0.57
McIntosh	Number occupied territories	11	10	13	12	14	11	11	11.71	11
	% nest success	82.0	80.0	77.0	83.0	71.0	91.0	83.0	81.00	82.0
	Brood size	1.56	1.63	1.30	1.40	1.40	1.60	1.30	1.46	1.20
	Productivity	1.27	1.30	1.00	1.17	1.00	1.45	1.08	1.18	1.00

Supplemental Table 2 Annual bald eagle nest survey data for coastal counties in Georgia, USA (2015-2022). A highly pathogenic influenza A virus outbreak was first documented in January of 2022 in this region, corresponding to bald eagle nesting season. Number of occupied nesting territories denotes count of nesting ranges containing evidence of breeding eagle interest, as assessed with aerial surveys; annual brood size is defined as mean number of fledglings produced per successful nest; annual productivity is calculated as mean number of fledglings per occupied nest territory.

County	Nest metrics	2016	2017	2018	2019	2020	2021	6 yr avg	2022
Brevard	Number occupied nests	2	5	15	20	22	31	15.83	39
	% nest success	100.0	100.0	86.7	70.0	81.8	80.6	86.50	41.0
	Brood size	2.00	1.80	1.77	1.43	1.78	1.68	1.74	1.63
	Productivity	2.00	1.80	1.53	1.00	1.45	1.35	1.52	0.67
Duval	Number occupied nests	14	15	19	25	24	24	20.17	26
	% nest success	78.6	66.7	84.2	80.0	95.8	66.7	78.70	88.5
	Brood size	1.73	1.70	1.88	1.75	1.52	1.69	1.71	1.57
	Productivity	1.36	1.13	1.58	1.40	1.46	1.13	1.34	1.38
Hillsborough	Number occupied nests	26	29	27	27	31	35	29.17	42
	% nest success	73.1	72.4	81.5	85.2	77.4	80.0	78.30	66.7
	Brood size	1.58	1.67	1.77	1.48	1.83	1.46	1.63	1.68
	Productivity	1.15	1.21	1.44	1.26	1.42	1.17	1.28	1.12
Seminole	Number occupied nests	9	22	24	22	27	34	23.00	34
	% nest success	88.9	68.2	79.2	86.4	88.9	85.3	82.80	85.3
	Brood size	1.25	1.47	1.53	1.63	1.58	1.59	1.51	1.62
	Productivity	1.11	1.00	1.21	1.41	1.41	1.35	1.25	1.38

Supplemental Table 3 Annual bald eagle nest survey data for early outbreak counties in Florida, USA (2016-2022). A highly pathogenic influenza A virus outbreak was first documented in January of 2022 in this region, corresponding to peak bald eagle nesting season. Number of occupied nests denotes nests monitored by EagleWatch team members that displayed evidence of breeding eagle interest; brood size is defined as mean number of fledglings produced per successful nests; productivity is defined as mean number of fledglings per occupied nests. Metrics for the 2022 season were extracted from database version 2022-05-19. 6 year averages for nesting metrics were calculated by treating each season (2016-2021) equally despite interannual variations in the total number of nests monitored by EagleWatch team members; this thus represents a general historical index for contextualizing the 2022 season results.

Legend - Supplementary Videos

Supplementary Video 1

Adult bald eagle with highly pathogenic avian influenza exhibiting clinical signs of disorientation, head shaking, and impaired mobility on March 17, 2022 in Pamlico County, North Carolina, USA just outside of the town of Oriental. Video courtesy of Chris Kent, District Wildlife Biologist, North Carolina Wildlife Resources Commission.

Supplementary Video 2

Adult bald eagle held in captivity with clinical signs of abnormal head positioning and movements attributed to highly pathogenic avian influenza on April 4, 2022 under the veterinary care of Donna Craig, DVM, CHPV. Video courtesy of Donna Craig of the Outback Mobile Veterinary Service in Melbourne, Florida.
